# Hospitalization rate of respiratory syncytial virus‐associated acute lower respiratory infection among young children in Suzhou, China, 2010–2014

**DOI:** 10.1111/irv.12958

**Published:** 2022-01-05

**Authors:** Shaolong Ren, Ting Shi, Wei Shan, Si Shen, Qinghui Chen, Wanqing Zhang, Zirui Dai, Jian Xue, Tao Zhang, Jianmei Tian, Genming Zhao

**Affiliations:** ^1^ Department of Epidemiology, School of Public Health Fudan University, Key Laboratory of Public Health Safety, Ministry of Education Shanghai China; ^2^ Soochow University Affiliated Children's Hospital Suzhou China

**Keywords:** acute lower respiratory infection, children, China, hospitalization rate, respiratory syncytial virus

## Abstract

**Background:**

There is a limited amount of data in China on the disease burden of respiratory syncytial virus‐ (RSV) associated acute lower respiratory infection (ALRI) among young children. This study aimed to estimate the hospitalization rate of RSV‐associated ALRI (RSV‐ALRI) among children aged 0–59 months in Suzhou, China.

**Methods:**

All cases from children hospitalized with ALRI who were aged 0–59 months in Suzhou University Affiliated Children's Hospital during January 2010 to December 2014 were retrospectively identified. Detailed diagnosis and treatment data were collected by reviewing each individual's medical chart. In accordance with the World Health Organization (WHO) influenza disease burden estimation, the hospitalization rate of RSV‐ALRI among children aged 0–59 months in Suzhou, China, was then estimated.

**Results:**

Out of the 28,209 ALRI cases, 19,317 (68.5%) were tested for RSV, of which the RSV positive proportion was 21.3% (4107/19,317). The average hospitalization rate of RSV‐ALRI for children aged 0–59 months was 14 (95% confidence interval [CI]:14–14)/1000 children years, and that for children aged 0–5, 6–11, 12–23, and 24–59 months were 70 (95% CI: 67–73), 31 (95% CI: 29–33), 11 (95% CI: 10–12), and 3 (95% CI: 3–3)/1000 children years, respectively.

**Conclusion:**

A considerable degree of RSV‐ALRI hospitalization exists among children aged 0–59 months, particularly in those under 1 year of age. Therefore, an effective monoclonal antibody or vaccine is urgently needed to address the substantial hospitalization burden of RSV infection.

## 1 INTRODUCTION

Acute lower respiratory tract infection (ALRI) remains one of the global leading causes of morbidity and mortality in children under 5 years of age.^1^, ^2^, ^3^ Common pathogens that give rise to ALRI in children include *Streptococcus pneumoniae*, *Haemophilus influenzae* type b, respiratory syncytial virus (RSV), and influenza virus. RSV is one of the most frequently encountered respiratory viruses that causes childhood ALRI.^2^, ^4^, ^5^ It has been estimated that RSV caused 33.1 million new cases of ALRI in children under 5 years of age in 2015, resulting in approximately 3.2 million hospitalizations and 59,600 in‐hospital deaths.^6^

Although a number of vaccines against RSV are currently under development, with some undergoing advanced stages of clinical trials, a licensed vaccine to prevent RSV infection has yet to be produced. The only prophylaxis currently approved by the Food and Drug Administration (FDA) is the monoclonal antibody palivizumab. However, palivizumab is only recommended for the prevention of serious disease in high‐risk infants, though its high costs hamper its widespread use.^7^, ^8^, ^9^ Therefore, estimating the disease burden of RSV infection is of great importance in terms of vaccine development and the prioritization of research funding.

Several studies have reported the prevalence and clinical characteristics of RSV infection among children in China.^10^, ^11^, ^12^, ^13^ However, research on the hospitalization burden of RSV‐ALRI among this population has not been thoroughly conducted. Hence, accurate estimates of disease burden due to RSV infection are necessary in order to bolster the development of vaccines and serve as guidance for future research priorities. Accordingly, by adopting the World Health Organization (WHO) method of influenza disease burden estimation,^14^, ^15^ this study attempts to estimate the hospitalization rate of RSV‐ALRI among children aged 0–59 months in Suzhou, China.

## 2 METHODS

### 2.1 Study site

**FIGURE 1:**
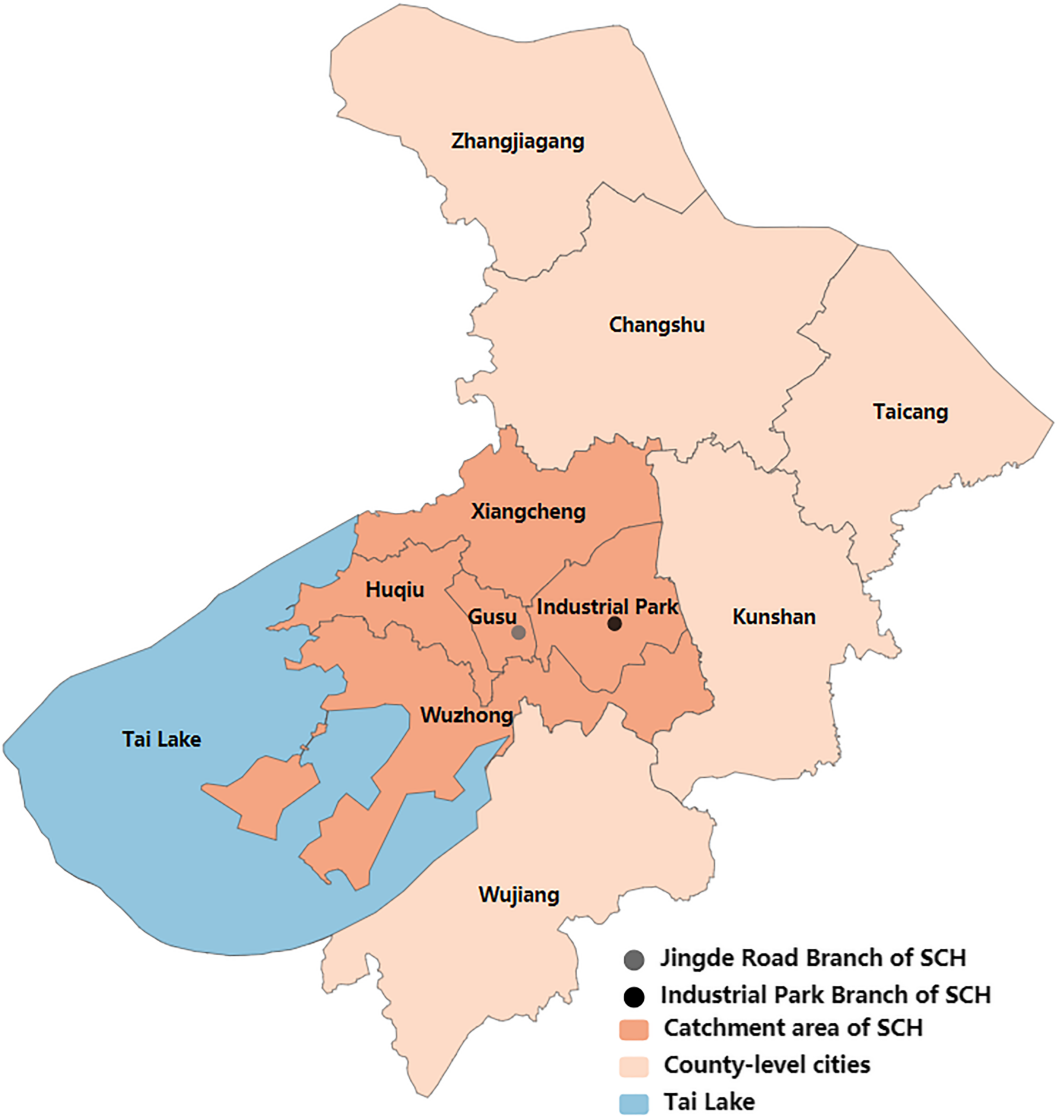
Geographic location and catchment area of SCH. SCH, Suzhou University Affiliated Children's Hospital

This retrospective study was conducted at Suzhou University Affiliated Children's Hospital (SCH). Suzhou is located in Jiangsu Province, southeast China, which had a population of 10 million in 2018. It has jurisdiction over five municipal districts (Gusu, Wuzhong, Huqiu, Xiangcheng, and Industrial Park) and five county‐level cities. SCH is a unique tertiary hospital for children in Suzhou and is composed of two branches: the Jingde Road Branch in the Gusu district and the Industrial Park Branch in the Industrial Park district. According to a previous study, the catchment area of SCH was defined to be the five municipal districts in Suzhou^16^ (Figure 1).

### 2.2 Data collection

ALRI was defined according to the International Classification of Disease 10th revision (ICD‐10) and discharge diagnosis codes J09‐J18 (influenza and pneumonia) and J20‐J22 (other ALRIs). Initially, all eligible hospitalized ALRI cases in SCH during January 2010 to December 2014 were retrospectively identified through the Hospital Information System. The inclusion criteria were (1) admission date was between January 1, 2010, and December 31, 2014; (2) patients were aged <5 years; (3) patients resided within the catchment area of SCH; (4) ICD‐10 disease codes for discharge diagnosis included J09‐J18 (influenza and pneumonia) or J20‐J22 (other ALRIs); and (5) patients were not admitted to hospital for the same disease within 30 days prior to admission. The following variables were exported from the Hospital Information System: admission number, date of birth, gender, address, date of admission, date of discharge, discharge diagnoses (ICD‐10 codes), and medical insurance status.^17^

**FIGURE 2:**
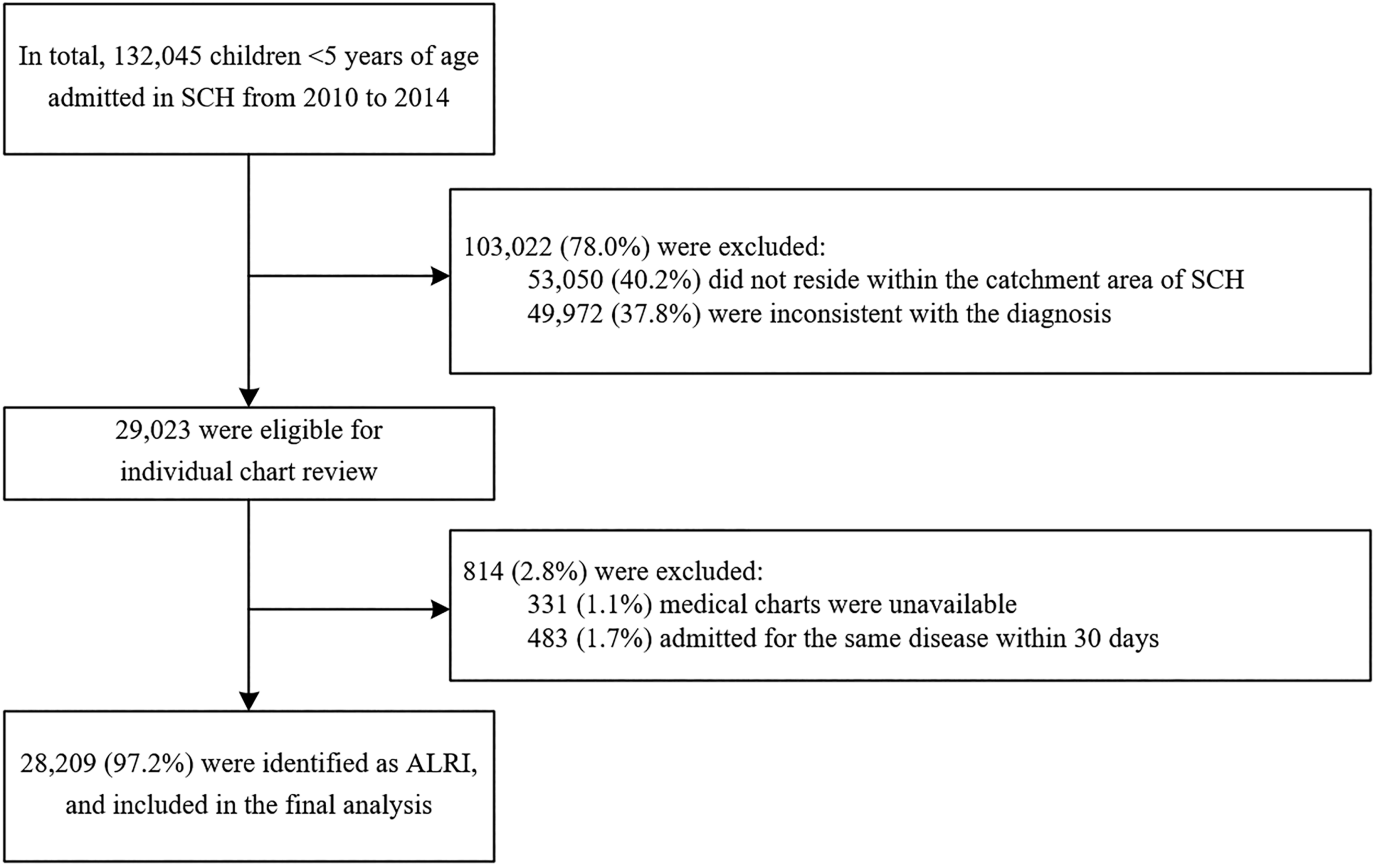
Enrollment of 28,209 hospitalized ALRI children aged 0–59 months. ALRI, acute lower respiratory infection; SCH, Suzhou University Affiliated Children's Hospital

Trained investigators were then recruited in order to extract the corresponding information from the individual medical chart of the identified ALRI cases. The variables collected in this step covered clinical manifestation, physical signs, biochemical testing results, pathogenic testing results, and therapeutic measures. RSV was detected using a direct immunofluorescence assay (D3 Ultra DFA respiratory virus screening and identification kit, Athens, Ohio, USA). In brief, smears of exfoliated cells were air dried, which was followed by cold acetone fixation. Then, 10 μl of monoclonal fluorescent antibodies against RSV was added to the smears. Specimens were incubated for 30 min in the dark at 37°C and were then subject to a phosphate buffer saline (PBS, pH 7.4) wash. Slides were examined under a fluorescence microscope (excitation wavelength = 488 nm), where a slide with five or more RSV inclusion bodies was considered to be RSV positive.^18^ Finally, 28,209 eligible ALRI children were screened out and included in the final analysis (Figure 2).

Referring to the definition of RSV epidemic period adopted by the U.S. Centers for Disease Control and Prevention,^19^, ^20^ the RSV epidemic period was defined as months in which the RSV positive rate exceeded 10% in the seasonal analysis.

### 2.3 Estimating the catchment population of SCH

According to our Healthcare Admission Survey (HAS) that was conducted in 2011, the hospital admissions for children under 5 years old in SCH accounted for 67.7% of the total hospital admissions in all medical institutions within the catchment area of SCH.^17^ Therefore, the annual catchment population of SCH by age group and gender was calculated as

Annual catchment population ofSCHbyagegroup and gender=67.7%×annual population of catchment areabyagegroup and gender.

The annual populations of catchment area by age group (0–5, 6–11, 12–23, and 24–59 months) and gender were obtained from the immunization program database of Suzhou Center for Disease Control and Prevention, which covers all residents in the aforementioned age groups.

### 2.4 Estimating RSV‐associated hospitalized ALRI cases

The monthly RSV positive proportion was first calculated by dividing the number of RSV positive hospitalized ALRI cases by the number of hospitalized ALRI cases tested for RSV:

$$\text{Monthly}\;\mathrm{RSV}\;\text{positive proportion}=\frac{\text{Monthly number of}\;\mathrm{RSV}\;\text{positive hospitalized ALRI cases}}{\text{Monthly number of hospitalized ALRI cases tested for}\;\mathrm{RSV}}.$$

It was assumed that the monthly RSV positive proportion of hospitalized ALRI children who were not tested for RSV would be similar to that of children within the same age group and gender stratum who were tested. Under this assumption, the monthly number of RSV‐associated hospitalized ALRI cases by age group and gender was calculated as

MonthlyRSV−associated hospitalized ALRI casesbyagegroup and gender=Monthly number ofRSVpositive hospitalized ALRI casesbyage group and gender+Monthly number of hospitalized ALRI cases untested forRSVbyagegroup and gender×α×MonthlyRSVpositive proportionbyagegroup and gender,

$$\text{where}\;\alpha =\frac{\mathrm{RSV}\;\text{positive proportion of hospitalized ALRI cases untested for}\;\mathrm{RSV}}{\mathrm{RSV}\;\text{positive proportion of hospitalized ALRI cases tested for}\;\mathrm{RSV}}=1.$$

The number of annual RSV‐associated hospitalized ALRI cases was calculated as

$$\text{Annual}\;\mathrm{RSV}-\text{associated hospitalized ALRI cases}\;\mathrm{by}\;\mathrm{age}\;\text{group and gender}=\sum_{\text{month}=1}^{12}\text{Monthly}\;\mathrm{RSV}-\text{associated hospitalized ALRI cases}\;\mathrm{by}\;\mathrm{age}\;\text{group and gender}.$$

### 2.5 Estimating the hospitalization rate of RSV‐associated ALRI

The annual hospitalization rate of RSV‐associated ALRI cases was then calculated as

$$\text{Annual hospitalization rate of}\;\mathrm{RSV}-\text{associated ALRI}\;\mathrm{by}\;\mathrm{age}\;\text{group and gender}=\frac{\text{Annual}\;\mathrm{RSV}-\text{associated hospitalized}\;\text{ALRI cases}\;\mathrm{by}\;\mathrm{age}\;\text{group and gender}}{\text{Annual catchment population}\;\mathrm{by}\;\mathrm{age}\;\text{group and gender}}\times 1000.$$

The confidence intervals (CIs) for the hospitalization rate were calculated according to the standard methods in the WHO guideline.^14^, ^15^

### 2.6 Sensitive analysis

In clinical practice, physicians may likely select suspected RSV infections cases when conducting RSV test. Hence, such a nonrandom test strategy may incur selection bias and affect the accuracy of the estimation. In order to address this, the characteristics between ALRI children who were not tested for RSV and in those who were tested were compared, after which the hospitalization rate of RSV‐ALRI was estimated in the following two additional conservative scenarios. In the first scenario, the monthly RSV positive proportion of hospitalized ALRI children who were not tested for RSV was assumed to be half of the children within the same age group and gender stratum who were tested, namely, α=0.5. In the second scenario, it was assumed that no RSV positive cases were present in the hospitalized ALRI cases untested for RSV, namely, α=0.

### 2.7 Statistical analysis

Categorical variables were presented as numbers or percentages. Chi‐square test was used to compare the RSV positive proportion between groups. A *P*‐value of <.05 was considered to be statistically significant. Data analyses were performed using R version 4.1.0 (R Foundation for Statistical Computing, Vienna, Austria).

### 2.8 Ethics statement

This study was conducted in accordance with internationally recognized standards for ethnical research and was approved by the Institute Review Board in the School of Public Health at Fudan University. Because this study was retrospective and utilized medical record data with no patient contact or collection of personal data, taking informed consent was not necessary.

## 3 RESULTS

### 3.1 Characteristics of study population

A total of 28,209 hospitalized cases were included in the final analysis, which consisted of 17,596 (62.4%) males and 10,613 (37.6%) females. The numbers of hospitalized ALRI children aged 0–5, 6–11, 12–23, and 24–59 months were found to be 9088 (32.2%), 5965 (21.1%), 5860 (20.8%), and 7296 (25.9%), respectively (Table 1).

**TABLE 1:** Basic characteristics and RSV testing results of hospitalized ALRI cases from 2010 to 2014

Characteristics	Number of ALRI cases (%)	ALRI tested for RSV		RSV positive		χ^2^ ^a^	*P*‐value
		N	%	N	%		
Year						202.0	<.001
2010	5311 (18.8)	2806	52.8	459	16.4		
2011	5666 (20.1)	3500	61.8	959	27.4		
2012	6096 (21.6)	4649	76.3	948	20.4		
2013	5539 (19.6)	4088	73.8	684	16.7		
2014	5597 (19.8)	4274	76.4	1057	24.7		
Gender						5.9	.015
Female	10,613 (37.6)	7111	67	1445	20.3		
Male	17,596(62.4)	12,206	69.4	2662	21.8		
Age (months)						749.3	<.001
0–5	9088 (32.2)	6877	75.7	2099	30.5		
6–11	5965 (21.1)	4187	70.2	935	22.3		
12–23	5860 (20.8)	3801	64.9	635	16.7		
24–59	7296 (25.9)	4452	61	438	9.8		
MedicaI insurance						59.1	<.001
Yes	13,175 (46.7)	8628	65.5	1617	18.7		
No	15,034 (53.3)	10,689	71.1	2490	23.3		
Premature^b^ ^,^ ^c^						3.3	.069
Yes	1754 (6.2)	1235	70.4	288	23.3		
No	25,557 (90.6)	17,782	69.6	3758	21.1		
Missing	898 (3.2)	300	33.4	61	20.3		
Congenital heart disease^c^						7.4	.007
Yes	1094 (3.9)	727	66.5	184	25.3		
No	26,941 (95.5)	18,526	68.8	3911	21.1		
Missing	174 (0.6)	64	36.8	12	18.8		
Total	28,209	19,317	68.5	4107	21.3		

Abbreviations: ALRI, acute lower respiratory infection; RSV, respiratory syncytial virus.

^a^ Chi‐square test for RSV positive rate.

^b^ Less than 37 weeks gestational age.

^c^ Missing data were excluded in Chi‐square test.

### 3.2 RSV positive proportion

Among the 28,209 ALRI cases, 19,317 (68.5%) were tested for RSV, for which the total RSV positive proportion was 21.3% (4107/19,317). The RSV positive proportion was found to be the highest in ALRI children aged 0–5 months (30.5%) but decreased to 22.3%, 16.7%, and 9.8% for children aged 6–11, 12–23, and 24–59 months, respectively. The RSV positive proportion in males was slightly higher than that in females (21.8% vs. 20.3%; *P* = .015). Meanwhile, the RSV positive proportion of ALRI children who had a history of congenital heart disease was shown to be higher compared with children without the disease (25.3% vs. 21.1%; *P* = .007). The RSV positive proportion of hospitalized ALRI cases varied from year to year (*P* < .001), in which higher RSV positive proportions were observed in 2011 (27.4%) and 2014 (24.7%) (Table 1).

### 3.3 Seasonal trend of RSV infection

**FIGURE 3:**
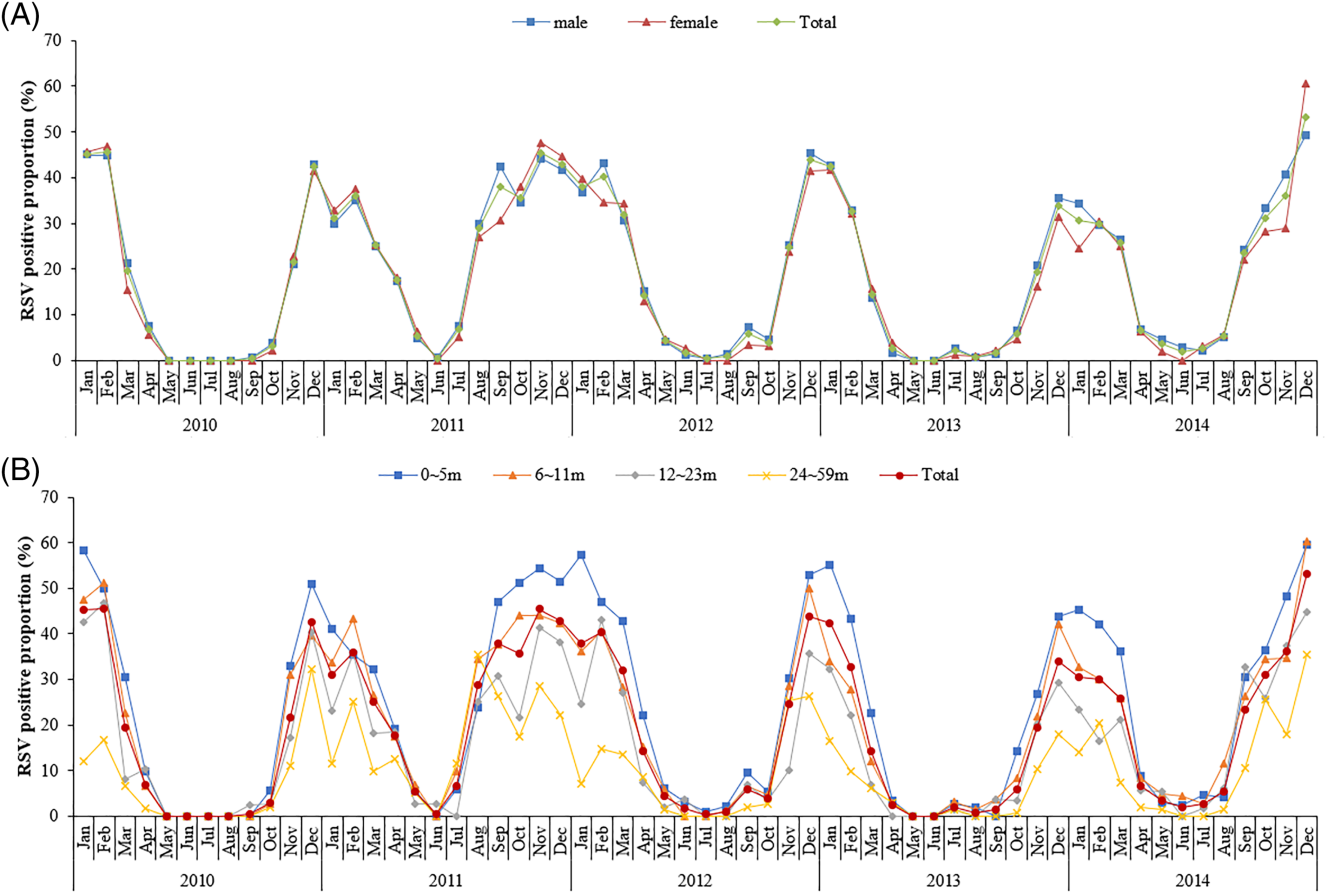
Monthly RSV positive proportion of hospitalized ALRI cases in SCH from 2010 to 2014 by gender or age group. (A) By gender. (B) By age group. ALRI, acute lower respiratory infection; RSV, respiratory syncytial virus; SCH, Suzhou University Affiliated Children's Hospital

The RSV positive proportion in hospitalized ALRI cases was observed to be highest in December 2014 (50.2%) and lowest in May to August 2010 and May to June 2013 (0%). The annual RSV epidemic in Suzhou generally started in November, peaked in December, and ended in March of the following year; however, the RSV epidemic started earlier in August and September in 2011 and 2014, respectively. The start time and duration of RSV epidemic varied annually (Figure 3).

### 3.4 Hospitalization rate

From 2010 to 2014, the RSV‐ALRI hospitalization rate of children aged 0–59 months in Suzhou was found to be 14 (95% CI: 14–14)/1000 children years, including 17 (95% CI: 16–18)/1000 children years for males and 10 (95% CI: 9–11)/1000 children years for females. Moreover, the RSV‐ALRI hospitalization rate was shown to be the highest in children aged 0–5 months, namely, 70 (95% CI: 67–73)/1000 children years, including 88 (95% CI: 84–92)/1000 children years for males and 51 (95% CI: 47–55)/1000 children years for females. The RSV‐ALRI hospitalization rate in 2011 and 2014 was higher than that in other years (Tables 2 and S1).

**TABLE 2:** Annual RSV‐ALRI hospitalization rate of children aged 0–59 months in Suzhou during 2010–2014 (per 1000 children years, 95% CI)

Year	Gender	0–11 months			12–23 months	24–59 months	0–59 months
		0–5 months	6–11 months	Total			
2010	Female	43 (34–52)	18 (12–24)	30 (25–35)	9 (6–12)	3 (2–4)	10 (9–11)
	Male	82 (71–93)	37 (29–45)	59 (52–66)	12 (9–15)	3 (2–4)	16 (14–18)
	Subtotal	64 (57–71)	28 (23–33)	45 (41–49)	12 (9–15)	3 (2–4)	13 (12–14)
2011	Female	65 (55–75)	40 (33–47)	51 (45–57)	10 (7–13)	5 (4–6)	15 (13–17)
	Male	121 (108–134)	59 (50–68)	88 (80–96)	19 (15–23)	5 (4–6)	24 (22–26)
	Subtotal	94 (86–102)	50 (44–56)	70 (65–75)	15 (13–17)	5 (4–6)	20 (19–21)
2012	Female	54 (46–62)	15 (11–19)	33 (29–37)	9 (7–11)	3 (2–4)	11 (10–12)
	Male	88 (79–97)	40 (34–46)	63 (58–68)	13 (10–16)	3 (2–4)	18 (17–19)
	Subtotal	71 (65–77)	28 (24–32)	49 (46–52)	11 (9–13)	3 (2–4)	15 (14–16)
2013	Female	48 (40–56)	15 (11–19)	30 (26–34)	5 (3–7)	2 (1–3)	8 (7–9)
	Male	71 (62–80)	30 (25–35)	49 (44–54)	10 (8–12)	2 (1–3)	12 (11–13)
	Subtotal	60 (54–66)	23 (20–26)	40 (37–43)	7 (6–8)	2 (2–2)	10 (9–11)
2014	Female	46 (40–52)	17 (13–21)	31 (27–35)	9 (7–11)	3 (2–4)	10 (9–11)
	Male	80 (72–88)	40 (34–46)	60 (55–65)	15 (12–18)	3 (2–4)	17 (16–18)
	Subtotal	63 (58–68)	29 (25–33)	46 (43–49)	12 (10–14)	3 (2–4)	13 (12–14)
Total	Female	51 (47–55)	20 (18–22)	35 (33–37)	8 (7–9)	3 (3–3)	10 (9–11)
	Male	88 (84–92)	41 (38–44)	63 (60–66)	14 (13–15)	3 (3–3)	17 (16–18)
	Subtotal	70 (67–73)	31 (29–33)	50 (48–52)	11 (10–12)	3 (3–3)	14 (14–14)

Abbreviations: ALRI, acute lower respiratory infection; CI, confidence interval; RSV, respiratory syncytial virus; RSV‐ALRI, RSV‐associated ALRI.

### 3.5 Sensitive analysis

Among the 28,209 ALRI cases, 19,317 were tested for RSV, of which the average RSV test proportion was found to be 68.5%. Meanwhile, the RSV test proportion of hospitalized ALRI cases varied annually (*P* < .001). Lower RSV test proportions were observed in 2010 (52.8%) and 2011 (61.8%), which was highest in ALRI children aged 0–5 months (75.7%) but decreased to 70.2%, 64.9%, and 61.0% for children aged 6–11, 12–23, and 24–59 months, respectively (Table 3).

**TABLE 3:** Comparison of ALRI cases tested for RSV with the untested in Suzhou University Affiliated Children's Hospital from 2010 to 2014

Characteristics	Number of ALRI cases, n (%)	Tested for RSV		χ^2^ ^a^	*P*‐value
		Yes, n (%)	No, n (%)		
Year				1125.4	<.001
2010	5311 (18.8)	2806 (52.8)	2505 (47.2)		
2011	5666 (20.1)	3500 (61.8)	2166 (38.2)		
2012	6096 (21.6)	4649 (76.3)	1447 (23.7)		
2013	5539 (19.6)	4088 (73.8)	1451 (26.2)		
2014	5597 (19.8)	4274 (76.4)	1323 (23.6)		
Gender				17.2	<.001
Female	10,613 (37.6)	7111 (67.0)	3502 (33.0)		
Male	17,596 (62.4)	12,206 (69.4)	5390 (30.6)		
Age (months)				449.5	<.001
0–5	9088 (32.2)	6877 (75.7)	2211 (24.3)		
6–11	5965 (21.1)	4187 (70.2)	1778 (29.8)		
12–23	5860 (20.8)	3801 (64.9)	2059 (35.1)		
24–59	7296 (25.9)	4452 (61.0)	2844 (39.0)		
Medical insurance				102.4	<.001
Yes	13,175(46.7)	8628 (65.5)	4547 (34.5)		
No	15,034 (53.3)	10,689 (71.1)	4345 (28.9)		
Premature^b^ ^,^ ^c^				0.5	.463
Yes	1754 (6.2)	1235 (70.4)	519 (29.6)		
No	25,557 (90.6)	17,782 (69.6)	7775 (30.4)		
Missing	898 (3.2)	300 (33.4)	598 (66.6)		
Congenital heart disease^c^				2.6	.106
Yes	1094 (3.9)	727 (66.5)	367 (33.5)		
No	26,941 (95.5)	18,526 (68.8)	8415 (31.2)		
Missing	174 (0.6)	64 (36.8)	110 (63.2)		
Total	28,209	19,317 (68.5)	8892 (31.5)		

Abbreviations: ALRI, acute lower respiratory infection; RSV, respiratory syncytial virus.

^a^ Chi‐square test for RSV test rate.

^b^ Less than 37 weeks gestational age.

^c^ Missing data were excluded in Chi‐square test.

**FIGURE 4:**
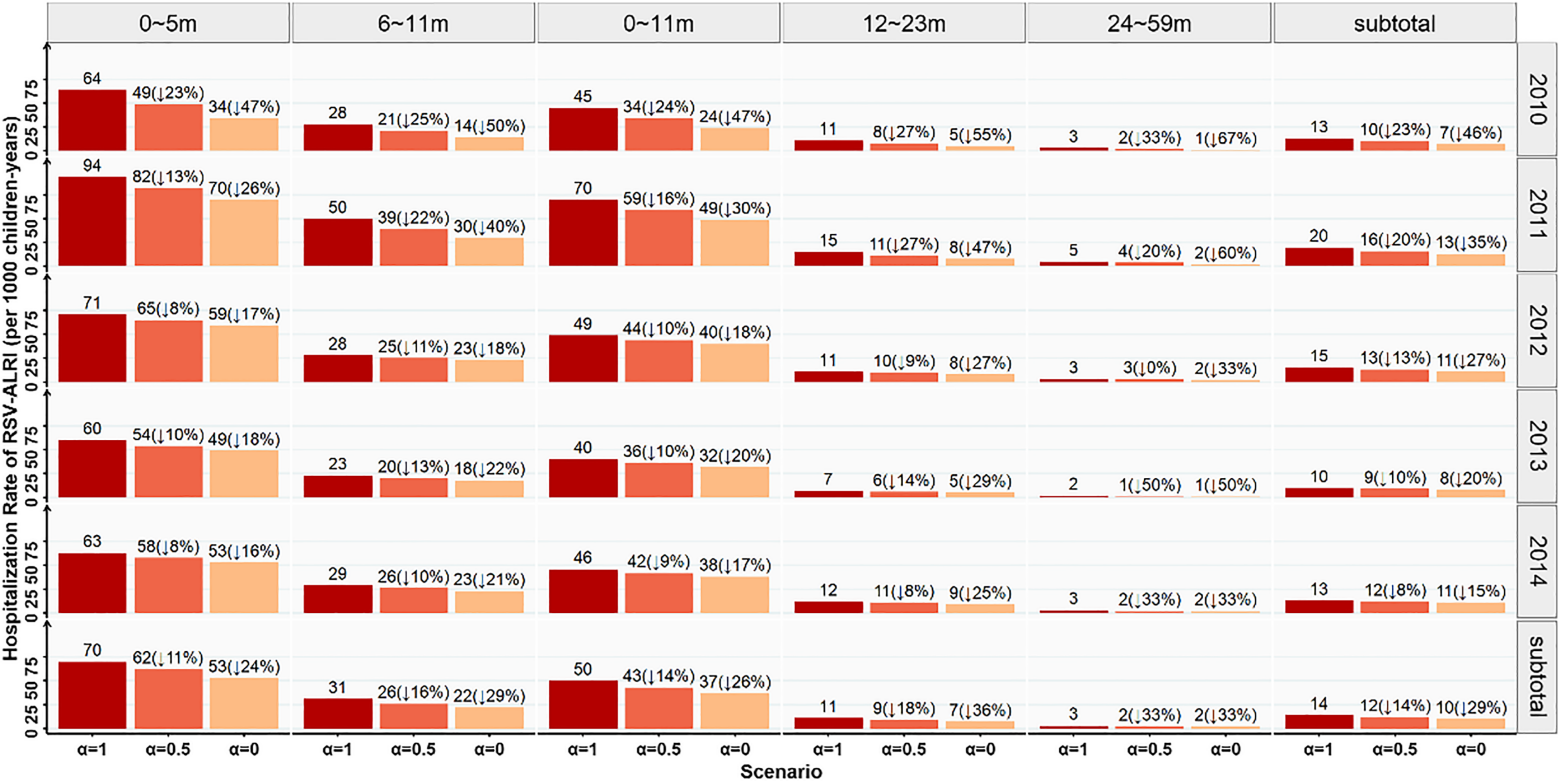
Hospitalized rate of RSV‐ALRI in SCH from 2010 to 2014 by age group under three scenarios. α: The ratio of positive proportion for hospitalized ALRI cases untested for RSV with those tested. (↓n%): The reduction percentage of hospitalization rate compared with the scenario when α=1. ALRI, acute lower respiratory infection; RSV, respiratory syncytial virus; RSV‐ALRI, RSV‐associated ALRI; SCH, Suzhou University Affiliated Children's Hospital

Compared with the scenario when α=1, the average hospitalization rate of RSV‐ALRI for children aged 0–59 months in the scenario when α=0.5 dropped 14% to 12 per 1000 children years, and reductions in the percentages of annual age‐specific hospitalization rate varied from 8% to 25%. The average hospitalization rate of RSV‐ALRI for children aged 0–59 months in scenario when α=0 dropped 29% to 10 per 1000 children years, and the percentage reduction of annual age‐specific hospitalization rate varied from 15% to 55% (Figure 4).

### 3.6 Discussion

In light of the obtained findings, the average RSV‐ALRI hospitalization rate for children aged 0–59 months in Suzhou was found to be 14 (95% CI:14–14) per 1000 children years. Moreover, the hospitalization rate was noted to be highest in children aged 0–5 months, namely, 70 per 1000 children years, which then decreased with rising age to 31 (6–11 months), 11 (12–23 months), and 3 (24–59 months) per 1000 children years. In terms of absolute number, 51% (2099/4107) hospitalized RSV‐ALRI cases were observed to be aged <6 months, which was similar to the proportion (45%) reported by Shi et al.^6^ Thus, an effective vaccine for use in the pediatric or maternal population is urgently needed. As of September 2021, more than 30 RSV vaccines are under development, of which two maternal vaccines are in phase III trials.^21^ If the trials succeed, these vaccines may be able to address the substantial RSV burden present in children.

As mentioned in the WHO guidelines, adopting a nonrandom test strategy may result in bias of hospitalization rate estimations. This study's estimates of hospitalization rates were based on the counterfactual hypothesis that if the ALRI cases untested for RSV were also tested, they would have a similar RSV positive proportion with those who were actually tested, namely, α=1. It was found that both RSV positive proportion and RSV test proportion decreased with a rise in age, detection bias may exist. However, the hypothesis α=1 could not be falsified as the RSV test results of those untested were unavailable. Accordingly, conducting an age stratified analysis strategy and sensitive analysis may partially remedy this potential bias. The sensitive analysis showed that the hospitalization rate of RSV‐ALRI in 2010 and 2011 dropped by 20% to 46% for α=0.5 or α=0. This sharp decrease was a result of the low RSV test proportion present in the corresponding two years (52.8% in 2010 and 61.8% in 2011). In contrast, about three quarters of ALRI cases were tested for ALRI in 2012–2014; hence, the hospitalization rate in 2012–2014 only dropped by 8% to 27% in the sensitivity analysis. In terms of age, due to the high RSV test proportion (75.7%) for ALRI cases aged 0–5 months, the hospitalization rate dropped by only 11% and 24% for α=0.5 and α=0, respectively. Young children with a higher RSV burden were found to have a higher RSV test proportion, which ensured the relative stability of this study's estimates for total disease burden. The average hospitalization rate of RSV‐ALRI for children aged 0–59 months only dropped by 14% and 29% for α=0.5 and α=0, respectively. Even in the extremely conservative scenario ( α=0), supposing that there were no RSV positive cases in hospitalized ALRI cases untested for RSV, the hospitalization rate of RSV‐ALRI for children aged 0–59 months remained at 10 per 1000 children years, which was 1.4 times greater than the hospitalization rate of influenza–severe acute respiratory infection (SARI) for children in Suzhou (7 per 1000 children years).^22^

The RSV positive proportion in the present study was found to be consistent with another retrospective study that was conducted in Wenzhou, China, using similar inclusion criteria.^23^ However, it was higher than three other studies, in which the RSV positive proportion varied from 8% in 0–5 years to 22% in children <6 months.^24^, ^25^, ^26^ Apart from differences in region and time, this study's diverse inclusion criteria may have also contributed to the observed disparity. In contrast to this study, the other three studies used the WHO definition of severe acute respiratory infection, in which fever (≥38°C) was an inclusion criterion. We screened ALRI cases that met the fever requirement and then analyzed the filtered data. In doing so, the RSV positive proportion for children aged 0–5 months was shown to decline significantly from 30.5% to 23.1% (Tables S2 and S3). Notably, as half of the hospitalized ALRI cases aged 0–59 months and four fifths hospitalized ALRI cases aged 0–5 months did not have fever (Table S4), including fever into the inclusion criteria may result in missing a substantial number of ALRI cases without fever. After including fever into the criteria, the hospitalization rate of RSV‐ALRI (with fever) for children aged 0–59 months dropped by 57% to 6 per 1000 children years and by 84% to 11 per 1000 children years for children aged 0–5 months (Figure S1). As confirmed by several studies, case definitions that include fever in RSV surveillance would result in a proportion of missed cases, particularly among young infants.^4^, ^5^, ^27^ In this regard, employing broad ALRI inclusion criteria made this study less likely to miss RSV‐infected cases and yield more accurate estimates of RSV‐ALRI hospitalization rates.

We found few original studies from China can be compared with the present study's estimates on the RSV‐ALRI hospitalization rate. Shi et al estimated that the incidence rate of RSV‐ALRI in China for children younger than 5 years was 31.0 (uncertainly range 18.7–50.8) per 1000 children years in 2015.^6^ They also estimated that about 10% of RSV‐ALRI episodes were severe enough to necessitate hospital admission globally.^2^, ^6^ According to this proportion, the hospitalization rate of RSV‐ALRI for children younger than 5 years in China would be approximately 3.1 per 1000 children years, which was lower than this study's most conservative estimates (10 per 1000 children years) in the Suzhou area. Shi et al used a risk‐factor‐based model to estimate RSV‐ALRI episodes among young children in 132 developing countries. Hence, their estimates may represent the average RSV‐ALRI burden in China. In contrast, the hospitalization rate of RSV‐ALRI was estimated within a specific catchment population in Suzhou in the present study; hence, the corresponding findings may be more specific to Suzhou. As a well‐developed city in the Yangtze delta, higher accessibility of health services may result in Suzhou having a higher RSV‐ALRI hospitalization rate than the national average. Additional research is thus required in order to elucidate the population‐based burden of RSV‐ALRI among young children in China.

Globally, estimates of RSV‐associated hospitalization rates vary from region to region. Shi et al estimated the hospitalization rate of RSV‐ALRI in developing countries for children aged 0–5, 6–11, and 12–59 months were 20.2, 11.0, and 1.5 per 1000 children years, respectively.^6^ Prasad et al estimated that the RSV‐associated acute respiratory infection (ARI) hospitalization rate during 2012–2015 was 6.1 (95% CI: 5.8–6.4) per 1000 children <5 years old in Auckland.^28^ Moreover, a separate modeling study estimated that the average RSV‐attributable burden of hospitalization in the United Kingdom during 1997–2009 was 41.8 (<6 months), 12.7 (6–23 months), and 1.1 (2–4 years) per 100 children years.^29^ Although this study's estimates for RSV‐ALRI hospitalization rate in Suzhou were about 2–3 times higher than the above results, they were still comparable. The associated difference may reflect real gaps in disease burden between countries; however, differences in health care policy and health care seeking behavior of the population should not be ignored. In this study, the median length of hospital stay for RSV‐ALRI cases was found to be 7 days, which was 2–3 times more than the 2–3 days reported in other studies.^6^, ^28^ Cheaper and more accessible health care service in China can partially explain the difference in hospitalization rate. Discrepancies in study design, cases definition, and testing method can result in less comparability between corresponding studies and highlight the need for the establishment of a uniform global RSV surveillance system.^30^

Consistent with previous research,^12^, ^18^ the annual RSV epidemic period in Suzhou was generally from November to March of the following year. Premature RSV epidemics in 2011 and 2014 extended the epidemic periods in the two years, which consequently caused a higher RSV positive proportion and hospitalization rate. Notably, the hospitalization rate of RSV‐ALRI in 2011 (20/1000 children years) was about twice than that in 2013 (10/1000 children years), indicating that the duration of the epidemic period had a large influence on the annual hospitalization burden of RSV‐ALRI infection. Therefore, predicting the annual start time of an RSV epidemic and implementing available forms of immunoprophylaxis ahead of time may be helpful in reducing the disease burden of RSV infection. Numerous studies have reported the association between RSV activity and meteorological factors.^12^, ^18^, ^31^ Identifying specific meteorological indicators and developing a prediction model for the RSV epidemic period based on these indicators may serve as potential directions for future research.

This study has several limitations. First, the obtained estimates of hospitalization rates should be inferred upon cautiously as data were only used from a single hospital in estimating RSV‐ALRI hospitalization rates in Suzhou. The average market share of SCH for the hospitalization of children under 5 years old was 67.7%, which was applied so as to estimate the catchment population of SCH in the present study. However, the market share of hospital may actually vary by disease in light of the presence of diverse specialties among different hospitals. Using the average market share may result in an overestimation or underestimation of the ALRI‐specific catchment population of SCH. Accordingly, this study's estimates for RSV‐ALRI hospitalization rate could be underestimated or overestimated. Second, direct immunofluorescence assay (DFA) is not considered to be the gold standard for RSV detection. The accuracy of the DFA test may affect this study's estimation of the RSV‐ALRI hospitalization rate. Third, RSVs are prone to coinfected with other pathogens. Accordingly, not considering coinfection may lead to overestimations of the RSV‐ALRI hospitalization rate.

## 4 CONCLUSIONS

In conclusion, this study showed a considerable level of RSV‐ALRI hospitalization among children aged 0–59 months in Suzhou, particularly among those under 1 year of age. Accordingly, an effective monoclonal antibody or vaccine is urgently needed to address this substantial disease burden brought about by RSV infection.

## FUNDING

This study was supported by the Shanghai New Three‐year Action Plan for Public Health (Grant No. GWV‐10.1‐XK16) and the Investigator‐Initiated Studies Program of Merck Sharp & Dohme Corp Grant (#61457). The funding agencies do not have any role in study design, data collection, data analysis, or draft of manuscript.

## AUTHOR CONTRIBUTIONS

**Shaolong Ren:** Data curation; formal analysis; software; visualization. **Ting Shi:** Data curation. **Wei Shan:** Data curation; software. **Si Shen:** Data curation; validation. **Qinghui Chen:** Data curation. **Jian Xue:** Data curation. **Zirui Dai:** Data curation; validation. **Wanqing Zhang:** Data curation; validation. **Tao Zhang:** Conceptualization; methodology; project administration; supervision. **Jianmei Tian:** Conceptualization; methodology; project administration; resources. **Genming Zhao:** Conceptualization; methodology; resources; supervision.

### PEER REVIEW

The peer review history for this article is available at https://publons.com/publon/10.1111/irv.12958.

## Supporting information


**Table S1.** Annual hospitalization rate of RSV‐ALRI by age group in Suzhou, China (2010–2014)
**Table S2.** RSV positive proportion of hospitalized ALRI cases
**Table S3.** RSV positive proportion of hospitalized ALRI cases with fever
**Table S4.** Fever proportion of hospitalized RSV‐ALRI cases
**Figure S1.** Hospitalized rate of RSV‐ALRI (with fever) in SCH from 2010 to 2014 by age group under three scenarios. Fever: ≥38 °C. α: the ratio of positive proportion for hospitalized ALRI cases (with fever) untested for RSV with those tested. (↓n%): the reduction percentage of hospitalization rate compared with the scenario when α=1. Abbreviations: RSV, Respiratory syncytial virus; ALRI, Acute lower respiratory infection; RSV‐ALRI, RSV‐associated ALRI; SCH, Suzhou University Affiliated Children's Hospital.

## Data Availability

The data that support the findings of this study are available from the corresponding author upon reasonable request.
