# Supplementary material for: Hospitalization rate of respiratory syncytial virus‐associated acute lower respiratory infection among young children in Suzhou, China, 2010–2014
Source: Influenza Other Respir Viruses. 2022 Jan 5;16(4):789–99. doi: 10.1111/irv.12958 (PMC9178065; doi:10.1111/irv.12958)
Supplement: Supplementary file 1 — Table S1. Annual hospitalization rate of RSV‐ALRI by age group in Suzhou, China (2010–2014) Table S2. RSV positive proportion of hospitalized ALRI cases Table S3. RSV positive proportion of hospitalized ALRI cases with fever Table S4. Fever proportion of hospitalized RSV‐ALRI cases Figure S1. Hospitalized rate of RSV‐ALRI (with fever) in SCH from 2010 to 2014 by age group under three scenarios. Fever: ≥38 °C. α: the ratio of positive proportion for hospitalized ALRI cases (with fever) untested for RSV with those tested. (↓n%): the reduction percentage of hospitalization rate compared with the scenario when α=1. Abbreviations: RSV, Respiratory syncytial virus; ALRI, Acute lower respiratory infection; RSV‐ALRI, RSV‐associated ALRI; SCH, Suzhou University Affiliated Children's Hospital. [file IRV-16-789-s001.docx]

**Supplementary Table1. Annual hospitalization rate of RSV-ALRI by age group in Suzhou, China (2010-2014)**

| Year | Age group | RSV positive  ALRI cases(a) | RSV  negative  ALRI cases(b) | ALRI cases  untested  for RSV(c) | RSV-ALRI cases(α=1) (d=a+c*a/(a+b)) | RSV-ALRI cases(α=0.5) (e=a+c*0.5*a/(a+b)) | RSV-ALRI cases(α=0)  (f=a) | Catchment population  (g) | Hospitalization rate of RSV-ALRI (α=1)  (/1000 children-years) (h=d*1000/g) | Variance for  RSV-ALRI cases(α=1) (i) | Variance for hospitalization rate of RSV-ALRI(α=1) (j=i*1000^2^/g^2^) | Hospitalization rate of  RSV-ALRI (α=1) (95%CI) [h(h±1.96$\sqrt{\boldsymbol{j}}$)] | Hospitalization rate of  RSV-ALRI (α=0.5) (/1000 children-years) (k= e*1000/g) | Hospitalization rate of  RSV-ALRI (α=0) (/1000 children-years) (l= f*1000/g) |
| --- | --- | --- | --- | --- | --- | --- | --- | --- | --- | --- | --- | --- | --- | --- |
| 2010 | 0~5m | 230 | 678 | 702 | 427 | 327 | 230 | 6717 | 64 | 650 | 14 | 64(57~71) | 49 | 34 |
|  | 6~11m | 101 | 495 | 545 | 205 | 153 | 101 | 7362 | 28 | 348 | 6 | 28(23~33) | 21 | 14 |
|  | 0~11m | 331 | 1173 | 1247 | 632 | 480 | 331 | 14079 | 45 | 998 | 5 | 45(41~49) | 34 | 24 |
|  | 12~23m | 73 | 462 | 524 | 152 | 114 | 73 | 14179 | 11 | 274 | 1 | 11(9~13) | 8 | 5 |
|  | 24~59m | 55 | 712 | 734 | 112 | 81 | 55 | 40380 | 3 | 219 | 0 | 3(2~4) | 2 | 1 |
|  | subtotal | 459 | 2347 | 2505 | 896 | 675 | 459 | 68638 | 13 | 1491 | 0 | 13(12~14) | 10 | 7 |
| 2011 | 0~5m | 483 | 1000 | 514 | 647 | 564 | 483 | 6862 | 94 | 784 | 17 | 94(86~102) | 82 | 70 |
|  | 6~11m | 235 | 554 | 505 | 392 | 308 | 235 | 7885 | 50 | 560 | 9 | 50(44~56) | 39 | 30 |
|  | 0~11m | 718 | 1554 | 1019 | 1039 | 872 | 718 | 14747 | 70 | 1344 | 6 | 70(65~75) | 59 | 49 |
|  | 12~23m | 138 | 473 | 508 | 247 | 190 | 138 | 16690 | 15 | 397 | 1 | 15(13~17) | 11 | 8 |
|  | 24~59m | 103 | 514 | 639 | 214 | 154 | 103 | 43894 | 5 | 400 | 0 | 5(4~6) | 4 | 2 |
|  | subtotal | 959 | 2541 | 2166 | 1500 | 1216 | 959 | 75331 | 20 | 2141 | 0 | 20(19~21) | 16 | 13 |
| 2012 | 0~5m | 495 | 1147 | 336 | 602 | 548 | 495 | 8421 | 71 | 684 | 10 | 71(65~77) | 65 | 59 |
|  | 6~11m | 214 | 799 | 247 | 267 | 241 | 214 | 9475 | 28 | 318 | 4 | 28(24~32) | 25 | 23 |
|  | 0~11m | 709 | 1946 | 583 | 869 | 789 | 709 | 17896 | 49 | 1002 | 3 | 49(46~52) | 44 | 40 |
|  | 12~23m | 140 | 751 | 332 | 191 | 164 | 140 | 17254 | 11 | 250 | 1 | 11(9~13) | 10 | 8 |
|  | 24~59m | 99 | 1004 | 532 | 145 | 120 | 99 | 47687 | 3 | 202 | 0 | 3(2~4) | 3 | 2 |
|  | subtotal | 948 | 3701 | 1447 | 1205 | 1073 | 948 | 82837 | 15 | 1454 | 0 | 15(14~16) | 13 | 11 |
| 2013 | 0~5m | 365 | 910 | 321 | 446 | 405 | 365 | 7440 | 60 | 510 | 9 | 60(54~66) | 54 | 49 |
|  | 6~11m | 155 | 768 | 270 | 198 | 175 | 155 | 8735 | 23 | 243 | 3 | 23(20~26) | 20 | 18 |
|  | 0~11m | 520 | 1678 | 591 | 644 | 580 | 520 | 16175 | 40 | 753 | 3 | 40(37~43) | 36 | 32 |
|  | 12~23m | 106 | 766 | 365 | 153 | 129 | 106 | 21139 | 7 | 209 | 0 | 7(6~8) | 6 | 5 |
|  | 24~59m | 58 | 960 | 495 | 86 | 70 | 58 | 51484 | 2 | 123 | 0 | 2(2~2) | 1 | 1 |
|  | subtotal | 684 | 3404 | 1451 | 883 | 779 | 684 | 88798 | 10 | 1085 | 0 | 10(9~11) | 9 | 8 |
| 2014 | 0~5m | 526 | 1043 | 338 | 624 | 573 | 526 | 9854 | 63 | 694 | 7 | 63(58~68) | 58 | 53 |
|  | 6~11m | 230 | 636 | 211 | 284 | 256 | 230 | 9854 | 29 | 332 | 3 | 29(25~33) | 26 | 23 |
|  | 0~11m | 756 | 1679 | 549 | 908 | 829 | 756 | 19708 | 46 | 1026 | 3 | 46(43~49) | 42 | 38 |
|  | 12~23m | 178 | 714 | 330 | 231 | 204 | 178 | 18954 | 12 | 290 | 1 | 12(10~14) | 11 | 9 |
|  | 24~59m | 123 | 824 | 444 | 178 | 148 | 123 | 59413 | 3 | 246 | 0 | 3(2~4) | 2 | 2 |
|  | subtotal | 1057 | 3217 | 1323 | 1317 | 1181 | 1057 | 98075 | 13 | 1562 | 0 | 13(12~14) | 12 | 11 |
| subtotal | 0~5m | 2099 | 4778 | 2211 | 2746 | 2417 | 2099 | 39294 | 70 | 3322 | 2 | 70(67~73) | 62 | 53 |
|  | 6~11m | 935 | 3252 | 1778 | 1346 | 1133 | 935 | 43311 | 31 | 1801 | 1 | 31(29~33) | 26 | 22 |
|  | 0~11m | 3034 | 8030 | 3989 | 4092 | 3550 | 3034 | 82605 | 50 | 5123 | 1 | 50(48~52) | 43 | 37 |
|  | 12~23m | 635 | 3166 | 2059 | 974 | 801 | 635 | 88216 | 11 | 1420 | 0 | 11(10~12) | 9 | 7 |
|  | 24~59m | 438 | 4014 | 2844 | 735 | 573 | 438 | 242858 | 3 | 1190 | 0 | 3(3~3) | 2 | 2 |
|  | subtotal | 4107 | 15210 | 8892 | 5801 | 4924 | 4107 | 413679 | 14 | 7733 | 0 | 14(14~14) | 12 | 10 |

α: the ratio of RSV positive proportion for hospitalized ALRI cases untested for RSV with those tested. Abbreviations: RSV, Respiratory syncytial virus; ALRI, Acute lower respiratory infection; RSV-ALRI, RSV-associated ALRI.

**Supplementary Table 2.** RSV positive proportion of hospitalized ALRI cases

| Age group | RSV positive | RSV negative | Untested for RSV | RSV positive proportion (%) |
| --- | --- | --- | --- | --- |
| 0~5m | 2099 | 4778 | 2211 | 30.5 |
| 6~11m | 935 | 3252 | 1778 | 22.3 |
| 12~23m | 635 | 3166 | 2059 | 16.7 |
| 24~59m | 438 | 4014 | 2844 | 9.8 |

**Supplementary Table 3.** RSV positive proportion of hospitalized ALRI cases with fever

| Age group | RSV positive | RSV negative | Untested for RSV | RSV positive proportion (%) |
| --- | --- | --- | --- | --- |
| 0~5m | 314 | 1043 | 548 | 23.1 |
| 6~11m | 447 | 1739 | 1015 | 20.4 |
| 12~23m | 447 | 2146 | 1380 | 17.2 |
| 24~59m | 357 | 3159 | 2083 | 10.2 |

**Supplementary Table 4.** Fever proportion of hospitalized RSV-ALRI cases

| Age group | Fever status | | | Fever proportion (%) |
| --- | --- | --- | --- | --- |
|  | Yes | No | Missing |  |
| 0~5m | 1905 | 6999 | 184 | 21.4 |
| 6~11m | 3201 | 2691 | 73 | 54.3 |
| 12~23m | 3973 | 1818 | 69 | 68.6 |
| 24~59m | 5599 | 1635 | 62 | 77.4 |
| Total | 14678 | 13143 | 388 | 52.7 |


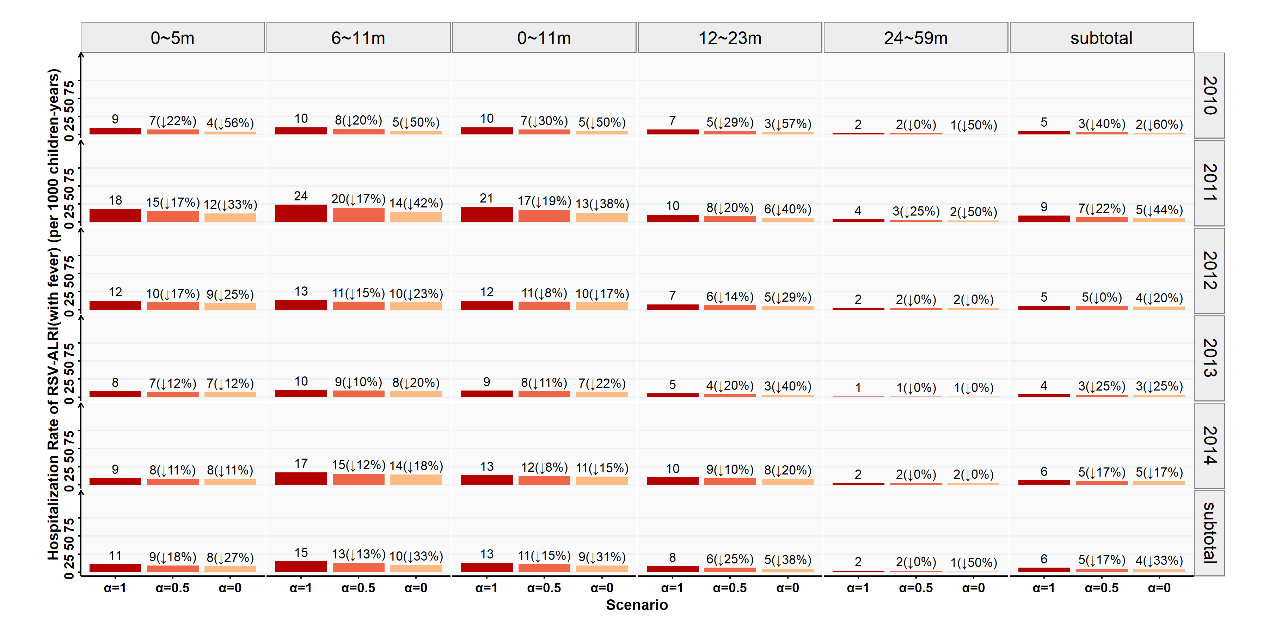


**Supplementary Figure 1.** Hospitalized rate of RSV-ALRI (with fever) in SCH from 2010 to 2014 by age group under three scenarios. Fever: ≥38℃. $\alpha$: the ratio of positive proportion for hospitalized ALRI cases (with fever) untested for RSV with those tested. (↓n%) : the reduction percentage of hospitalization rate compared with the scenario when $\alpha=1$. Abbreviations: RSV, Respiratory syncytial virus; ALRI, Acute lower respiratory infection; RSV-ALRI, RSV-associated ALRI; SCH, Suzhou University Affiliated Children’s Hospital.
